# Cutaneous Tuberculosis in the Modern Era: A Case of Lupus Vulgaris with Surgical Management and a Review of Clinical Spectrum, Diagnostic Challenges, and Malignant Potential

**DOI:** 10.3390/jcm15020702

**Published:** 2026-01-15

**Authors:** Klaudia Knecht-Gurwin, Iwona Chlebicka, Lukasz Matusiak, Zdzisław Woźniak, Andrzej Bieniek, Jacek C. Szepietowski

**Affiliations:** 1 University Center for General and Oncological Dermatology, Wroclaw Medical University, 50-367 Wroclaw, Poland; 2 Department of Dermato-Venereology, 4th Military Hospital, 50-981 Wroclaw, Poland; 3 Division of Dermatology, Venereology and Clinical Immunology, Faculty of Medicine, Wroclaw University of Science and Technology, 50-377 Wroclaw, Poland; 4 Faculty of Medicine and Faculty of Cosmetology, Calisia University, 62-800 Kalisz, Poland

**Keywords:** cutaneous tuberculosis, lupus vulgaris, *Mycobacterium tuberculosis*, anti-tubercular therapy, squamous cell carcinoma, chronic inflammation, surgical management

## Abstract

**Background/Objectives**: Cutaneous tuberculosis (CTB) represents a rare extrapulmonary manifestation of *Mycobacterium tuberculosis* infection, accounting for approximately 1–2% of all tuberculosis cases. Despite its low incidence, CTB remains diagnostically challenging due to its clinical polymorphism and resemblance to other granulomatous or neoplastic dermatoses. Among its variants, lupus vulgaris (LV) constitutes the most common and indolent form in regions of moderate tuberculosis endemicity. The present study aims to highlight the diagnostic complexity, management, and long-term outcomes of LV, emphasizing its potential for malignant transformation into squamous cell carcinoma (SCC). **Methods**: We present a detailed case of lupus vulgaris in a male patient with a prolonged disease course, refractory to initial empiric therapy, successfully managed through anti-tubercular therapy (ATT) followed by surgical excision. A review of the literature was conducted to contextualize this case within the broader clinical spectrum of CTB, with particular attention to epidemiology, histopathology, and complications, including SCC development. **Results**: The patient demonstrated significant clinical improvement following standard six-month ATT; however, residual fibrotic lesions required excision for definitive management. Literature review revealed that chronic LV lesions persisting for decades may undergo malignant transformation. Analysis of reported cases underscores the importance of vigilance and early surgical intervention in long-standing or atypical LV. **Conclusions**: Lupus vulgaris remains a clinically deceptive entity requiring multidisciplinary management. Early recognition, appropriate ATT, and surgical excision of residual or recalcitrant lesions are crucial to prevent complications, including carcinogenesis. Greater clinician awareness of CTB’s diverse presentations may reduce diagnostic delays and improve outcomes.

## 1. Introduction

Tuberculosis (TB) remains a formidable global health challenge, having recently reclaimed its position as the foremost cause of mortality from infectious diseases worldwide [1]. According to the World Health Organization (WHO), in 2023 an estimated 8.2 million individuals were newly diagnosed with TB—the highest annual incidence recorded since systematic global monitoring commenced in 1995—while approximately 1.25 million deaths were attributed to the disease in the same year [1,2]. Although pulmonary TB predominates, extrapulmonary forms constitute a substantial proportion, accounting for 20–30% of all active cases, and may involve virtually any anatomical site with diverse clinical sequelae [3]. Within this spectrum, cutaneous tuberculosis (CTB) represents an uncommon manifestation, occurring in approximately 1–2% of patients, yet it poses disproportionate diagnostic and therapeutic challenges [4]. CTB is frequently underrecognized, often masquerading as other dermatologic or infectious conditions, which contributes to considerable delays in establishing the correct diagnosis. The clinical spectrum of CTB is remarkably heterogeneous, ranging from verrucous plaques and ulcerations to nodular and cicatricial lesions. Lupus vulgaris (LV), the most common presentation in Europe and other regions of moderate TB endemicity, is a chronic, indolent form characterized by granulomatous inflammation, slowly progressive scarring, and, in long-standing cases, a potential for malignant transformation into squamous cell carcinoma (SCC) [5].

Given the ongoing global resurgence of tuberculosis and the increasing recognition of extrapulmonary forms, awareness of cutaneous TB remains crucial. This article presents a detailed case of lupus vulgaris, accompanied by a review of its clinicopathologic spectrum, management strategies, and oncogenic potential, emphasizing the importance of timely diagnosis and multidisciplinary care.

## 2. Clinical Spectrum of CTB

Cutaneous tuberculosis (CTB) represents a rare but clinically diverse manifestation of *Mycobacterium tuberculosis* infection, constituting approximately 1–2% of all tuberculosis cases worldwide. The cutaneous forms reflect a dynamic interplay between the pathogen’s virulence, the route of infection, and the host’s immune response. Depending on these factors, CTB may present as either paucibacillary or multibacillary disease, with clinical patterns ranging from indolent plaques to ulcerative, destructive lesions. Although uncommon, CTB remains of enduring clinical relevance, particularly in endemic regions and immunocompromised populations, as well as in developed countries where diagnostic unfamiliarity frequently leads to delays and misdiagnosis.

The classification of CTB traditionally relies on two main parameters: the mode of infection—exogenous inoculation, contiguous spread from an underlying tuberculous focus, or hematogenous dissemination—and the host’s degree of cell-mediated immunity. In individuals with robust immunity, the infection tends to remain localized, producing chronic, paucibacillary forms such as LV or tuberculosis verrucosa cutis (TVC). Conversely, patients with impaired immunity are predisposed to disseminated, multibacillary variants such as scrofuloderma or metastatic tuberculous abscesses. In addition, tuberculids-immunologically mediated eruptions triggered by distant mycobacterial antigens-represent a unique spectrum of hypersensitivity reactions rather than true infections. Despite being an ancient disease, CTB continues to perplex clinicians, often masquerading as fungal, bacterial, or neoplastic conditions, leading to significant diagnostic delay.

### 2.1. Lupus Vulgaris

The most emblematic manifestation of CTB in temperate climates is LV, a slowly progressive, chronic form that develops in previously sensitized hosts. LV, also known as tuberculosis luposa, is a chronic and indolent manifestation of cutaneous tuberculosis, characterized by its paucibacillary nature and progressive course. It typically arises in individuals with moderate to high levels of cell-mediated immunity to Mycobacterium tuberculosis and constitutes a major clinical form of CTB in countries with moderate tuberculosis endemicity [4,6,7,8]. Although once prevalent across Europe, the incidence of LV has declined markedly in high-income countries, yet it remains a public health concern in endemic areas. The condition exhibits a notable predilection for females, who are affected approximately two to three times more frequently than males, and spans all age groups [9]. LV commonly results from hematogenous, lymphatic, or contiguous spread from an internal focus of tuberculosis, although direct inoculation into the skin is also possible [10].

Clinically, it presents as slowly enlarging, erythematous to brownish-red plaques with central atrophy, most often localized to the head and neck region. On diascopy, these lesions exhibit a characteristic “apple-jelly” hue, attributable to granulomatous infiltration in the dermis [11].

Histopathologically, LV is defined by well-formed tuberculoid granulomas, with or without caseation necrosis, accompanied by a variable degree of epidermal hyperkeratosis or atrophy [12]. Due to its paucibacillary character, acid-fast bacilli are infrequently identified [13].

Diagnostic confirmation therefore often depends on molecular assays such as PCR, which offer higher sensitivity compared to conventional culture. While anti-tubercular therapy (ATT) is highly effective in resolving active infection, long-standing, untreated lesions may lead to irreversible tissue damage and, in rare cases, malignant transformation into squamous cell carcinoma (SCC) [13].

### 2.2. Scrofuloderma

Scrofuloderma represents the most common multibacillary form of CTB and typically develops through direct extension of an underlying tuberculous focus—most frequently infected lymph nodes, bone, or joints—into the overlying dermis [14]. It is most frequently seen in children and young adults in endemic regions, particularly in association with cervical lymphadenitis.

Clinically, scrofuloderma manifests as firm subcutaneous nodules that gradually soften and ulcerate, discharging caseous or purulent material through sinus tracts. The ulcers have irregular, undermined edges with violaceous borders. The healing process often leaves retractile scars and keloids, resulting in substantial cosmetic morbidity [3,14].

Histologically, the dermis shows tuberculoid granulomas with central caseation and abscess formation; acid-fast bacilli are usually demonstrable. Scrofuloderma is almost always culture-positive and thus serves as an important diagnostic source for confirming *M. tuberculosis* infection. Systemic involvement—most often pulmonary or skeletal tuberculosis—should always be investigated through imaging and microbiologic testing [15].

### 2.3. Tuberculosis Verrucosa Cutis

Tuberculosis verrucosa cutis (TVC), also known as warty tuberculosis, is a form of reinfection tuberculosis occurring in individuals with high immunity due to previous sensitization. It results from exogenous inoculation of mycobacteria into the skin of an individual with intact immunity [14].

Clinically, TVC presents as a solitary, verrucous, hyperkeratotic plaque with an irregular, serpiginous border, usually located on exposed areas such as the hands, knees, or buttocks. The lesion expands centrifugally and may resemble verruca vulgaris, deep fungal infection, or chromoblastomycosis [16].

Histopathologically, it is characterized by pseudoepitheliomatous hyperplasia, compact hyperkeratosis, and dermal granulomas composed of epithelioid cells and Langhans giant cells with minimal caseation. Acid-fast bacilli are rarely seen. The chronic course and verrucous morphology make TVC a common source of diagnostic confusion, necessitating histopathological confirmation [17].

### 2.4. Tuberculous Chancre

Tuberculous chancre, also termed primary inoculation tuberculosis, occurs in individuals without prior exposure or immunity to *M. tuberculosis*. It develops after direct inoculation of bacilli into abraded skin, most often in medical personnel, butchers, or laboratory workers.

Within 2–4 weeks of inoculation, a painless papule appears at the site of trauma, which evolves into an ulcer with undermined borders and a granular base. Regional lymphadenitis is a hallmark feature and may precede systemic dissemination if left untreated [14,18].

Histopathology initially shows a nonspecific suppurative infiltrate that evolves into classic tuberculoid granulomas with epithelioid cells and caseous necrosis. Acid-fast bacilli are typically abundant in early lesions. This form exemplifies the “primary complex” of CTB [19].

### 2.5. Tuberculosis Orificialis

Tuberculosis orificialis, or tuberculosis cutis orificialis, is an autoinoculation form of CTB that occurs in patients with advanced pulmonary, gastrointestinal, or genitourinary tuberculosis, often those who are immunocompromised. The organism spreads via expectoration, defecation, or urination to adjacent mucocutaneous junctions [20].

Clinically, it presents as painful, shallow ulcers with undermined edges located at the oral cavity, anus, or genitalia. Lesions may coalesce and produce marked discomfort, leading to impaired nutrition or secondary infection. Histopathology reveals caseating granulomas rich in acid-fast bacilli. Because of its strong association with disseminated tuberculosis, this manifestation often portends a poor prognosis [21].

### 2.6. Tuberculous Gumma and Miliary Cutaneous Tuberculosis

Tuberculous gumma, also known as metastatic tuberculous abscess, results from hematogenous spread of *M. tuberculosis* from a distant focus, often during periods of reduced cell-mediated immunity. It presents as multiple deep, cold abscesses that later ulcerate, releasing caseous material [14].

Cutaneous miliary tuberculosis, in contrast, represents widespread hematogenous dissemination of bacilli in severely immunocompromised patients or those with miliary systemic disease. Lesions are multiple, small, erythematous papules or pustules that may necrotize centrally. Both conditions carry high mortality and require prompt recognition and systemic therapy [14,15].

### 2.7. Tuberculids

Tuberculids are not true infections but rather hypersensitivity reactions to *M. tuberculosis* antigens in individuals with moderate to strong immunity and a distant focus of tuberculosis.

- Papulonecrotic tuberculid manifests as symmetrical, necrotic papules on the extensor surfaces of the limbs and trunk, often healing with varioliform scars. Histology reveals leukocytoclastic vasculitis with fibrinoid necrosis and rare granulomas; acid-fast bacilli are absent [22].
- Lichen scrofulosorum presents as tiny, perifollicular papules arranged in clusters, primarily in children and young adults. It is associated with underlying lymph node or bone tuberculosis [23].
- Erythema induratum of Bazin is a nodular, often ulcerative panniculitis seen predominantly in women, affecting the posterior legs. Histopathology demonstrates lobular panniculitis with granulomatous vasculitis [24].

These forms highlight the immunological spectrum of TB, where vigorous host responses paradoxically give rise to cutaneous inflammation in the absence of viable organisms. Table 1 summarizes the major clinicopathological forms of CTB, highlighting their etiopathogenesis, morphology, and diagnostic hallmarks.

### 2.8. Pathological Hallmarks and Diagnostic Considerations

Across all clinical variants, the histopathologic hallmark of CTB is the formation of tuberculoid granulomas composed of epithelioid histiocytes, Langhans giant cells, and peripheral lymphocytes, with variable degrees of caseous necrosis. The demonstration of acid-fast bacilli via Ziehl-Neelsen or Auramine-rhodamine staining is inconsistent, being more likely in multibacillary forms such as scrofuloderma or orificialis TB, and rarely in paucibacillary entities like LV or TVC. Molecular diagnostics, particularly polymerase chain reaction (PCR) targeting *M. tuberculosis* complex DNA, have revolutionized CTB diagnosis, providing rapid and sensitive confirmation even in paucibacillary cases. Culture, though less sensitive, remains the gold standard for species identification and drug-susceptibility testing. A complete diagnostic approach should integrate clinical morphology, histopathology, and molecular assays, along with radiologic evaluation to detect systemic involvement [14,18].

## 3. Case Presentation

A 72-year-old male with a history of hypertension, ischemic heart disease, and atrial fibrillation presented with chronic, non-healing skin lesions. The first lesion had appeared three years earlier on the back, was incised and drained, but recurred shortly after. Two additional lesions developed—one on the back and one on the left cheek. The lesions exhibited continuous sero-bloody discharge but were non-painful. Cultures were initially negative. A skin biopsy from the back showed chronic inflammation with hyperkeratosis and no evidence of malignancy. Dapsone therapy (50 mg/day) was initiated but proved ineffective.

**Figure 1:**
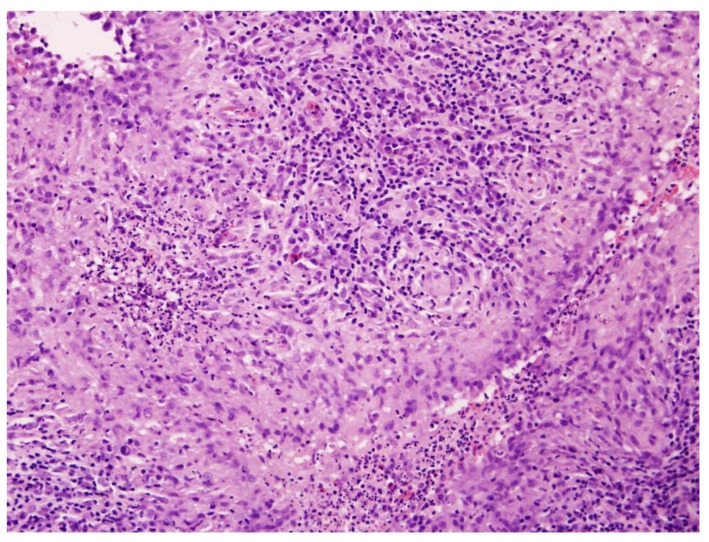
Histopathological image of cutaneous tuberculosis (H&E staining, ×100). The dermis contains well-formed tuberculoid granulomas composed of epithelioid histiocytes and Langhans-type multinucleated giant cells, surrounded by dense lymphoplasmacytic infiltrate. Caseation necrosis is absent, consistent with LV.

On admission, biopsies from the cheek and back were obtained. Histopathological examination of lesions revealed fragments of stratified squamous epithelium, possibly consistent with a ruptured epidermoid cyst, surrounded by a diffuse inflammatory infiltrate composed predominantly of lymphocytes, plasma cells, and histiocytes. Numerous palisading histiocytes formed granulomatous structures interspersed with scattered multinucleated Langhans-type giant cells. The dermis exhibited patchy fibrosis and areas of stromal homogenization, and involvement of the superficial subcutaneous tissue was evident. No caseation necrosis was present. The epidermis showed focal hyperkeratosis and acanthosis without evidence of epithelial dysplasia or malignant transformation. The histologic findings were consistent with, though not pathognomonic for, CTB (Figure 1). PCR confirmed the presence of *M. tuberculosis* DNA. Systemic evaluation was performed to assess for extracutaneous involvement. No clinical or radiologic evidence of pulmonary, lymph node, bone, or visceral tuberculosis was identified, and the disease was considered limited to the skin. A diagnosis of LV was made, and ATT (rifampicin 600 mg/day and isoniazid 300 mg/day) was initiated. The therapeutic regimen was prescribed following consultation with a tuberculosis specialist, with the choice of agents influenced by the patient’s concomitant medications and the potential drug interactions, and consisted of a 6-month course of combination therapy.

Following completion of the planned course of anti-tubercular therapy, treatment was discontinued after phthisiatric consultation and the patient was placed under observation. During a control visit one year later, residual cutaneous lesions and post-tuberculous scars were noted. Surgical excision of a residual scar was performed with informed consent, and the wound healed uneventfully without evidence of pathergy.

**Figure 2:**
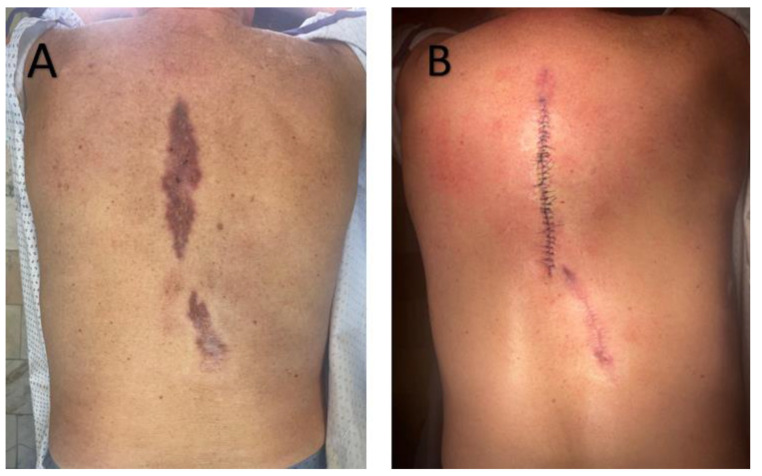
(**A**)—Sharply demarcated, irregular, brownish-red plaque with surface scaling on the back. (**B**)—Postoperative view following wide local excision of the lesion. Surgical sutures are visible, with no signs of local recurrence at follow-up.

Approximately six months later, the patient was re-admitted with an erythematous-infiltrated paraspinal lesion measuring about 20 cm, accompanied by a postoperative scar in the lumbar region (Figure 2). Surgical excision of a 10 cm interscapular scar and granulomatous tissue from the lumbar site was performed. At a six-month reassessment, no new lesions were detected, and only post-surgical scarring was present.

## 4. Discussion

LV, though infrequent today, poses a diagnostic and therapeutic challenge due to its indolent course, diverse morphology, and potential complications. In our case, the initial delay in diagnosis and lack of response to empiric therapy exemplify the difficulty in recognizing LV. Despite appropriate ATT and partial clinical improvement, residual inflammatory lesions with mild serous discharge persisted, prompting surgical excision as a combined therapeutic and diagnostic intervention that provided symptom relief and enabled comprehensive histopathological evaluation to exclude occult malignant transformation. This approach aligns with emerging perspectives in the literature. While ATT remains the cornerstone of treatment, surgical excision can be a valuable adjunct in recalcitrant cases or when residual scarring and tissue damage threaten function or aesthetics. Standard short-course chemotherapy (usually six months of rifampicin and isoniazid in drug-susceptible disease) achieves cure rates exceeding 90–95%, but relapse, though uncommon, has been reported. Some authors advocate for extended regimens of 9–12 months in extensive cutaneous disease to ensure complete sterilization. Importantly, multidrug-resistant TB (MDR-TB) has been described as a cause of LV, requiring second-line regimens of considerably longer duration, which increases the burden of treatment and the risk of toxicity. More importantly, chronic LV lesions—particularly those with long-standing ulceration and scarring—pose a recognized albeit rare risk of malignant transformation into SCC, with incidence rates reported between 0.5% and 10.5% [25,26]. To contextualize this risk, we reviewed published reports of SCC arising in LV, identified through a targeted narrative search of PubMed/MEDLINE, Embase, Web of Science, and Scopus using combinations of keywords such as “lupus vulgaris,” “cutaneous tuberculosis,” “squamous cell carcinoma,” “malignant transformation,” and “Marjolin ulcer.” Given the narrative nature of this review and the rarity of malignant transformation in LV publications were selected based on their clinical relevance and the availability of detailed clinicopathological data.

Analysis of the reported cases indicates that malignant transformation most often occurs after prolonged disease duration, frequently exceeding several decades, although more rapid progression has also been described, particularly in neglected or misdiagnosed lesions (Table 2). The majority of cases have occurred after prolonged disease duration, often exceeding several decades, and most frequently involved the head and neck. For instance, Yerushalmi et al. [27] described a 47-year-old Bedouin man with a 40-year history of LV of the posterior neck who developed moderately differentiated SCC with regional nodal spread; combined surgical resection, anti-TB therapy, and radiotherapy achieved local control without recurrence. Similarly, Gooptu et al. [28] reported a 64-year-old woman in the United Kingdom with facial LV irradiated decades earlier with a Finsen lamp, who subsequently developed SCC; excision resulted in durable cure. Multiple cases from endemic regions mirror these findings. In Romania, Pătrașcu et al. [29] described a 59-year-old woman with LV of the ear and cheek persisting for 57 years, which progressed to SCC with nodal metastasis; surgical excision and lymph node dissection were performed, with ongoing follow-up. From Poland, Zawirska et al. [30] reported a 65-year-old man with exfoliative LV of the cheeks of 40 years’ duration that transformed into SCC, successfully treated by surgery and anti-TB therapy.

More aggressive courses have also been noted. Wulff-Wösten et al. [31] described a 69-year-old German woman with a 55-year history of LV of the thigh, who developed SCC with lymphatic spread and ultimately died of metastatic disease despite multimodal treatment. In contrast, Erdem et al. [32] observed a 45-year-old Turkish man with a 20-year course of LV on the face and neck that transformed into well-differentiated SCC but was cured after excision and ATT.

Notably, malignant transformation has also been documented in younger patients and in the setting of multidrug-resistant TB. Kumaran et al. [33] reported a 34-year-old Indian man with MDR LV in the beard area who developed well-differentiated SCC after 10 years of disease; surgical excision and second-line anti-TB therapy achieved remission. Similarly, Miyake et al. [26] described a 71-year-old Japanese man with 60 years of untreated LV on the face who developed SCC, cured with excision and anti-TB therapy. Lin et al. [34] recently reported a 54-year-old Chinese man with a 20-year history of facial LV, in whom SCC was treated successfully with surgery, ATT, and adjunct photodynamic therapy.

Cases have also been observed on atypical sites. Chlebicka et al. [5] described a 62-year-old Polish woman with LV of the hand of only 5 years’ duration, who developed SCC confirmed by PCR for *M. tuberculosis*. Excision with a skin graft achieved complete remission and preserved hand function. Kanitakis et al. [35] documented a 70-year-old French woman with a 45-year history of facial LV who developed well-differentiated SCC, managed successfully with surgery.

Taken together, these reports demonstrate several recurring themes: malignant transformation of LV is strongly associated with long disease duration, chronic scarring, and head-and-neck localization, but can also occur at atypical sites and even after shorter disease intervals. Prognosis depends largely on early recognition and complete surgical excision; while most cases achieved cure, a minority suffered nodal metastasis or fatal dissemination. These observations reinforce the need for long-term vigilance and proactive management in persistent LV.

While direct evidence specifically linking cutaneous *M. tuberculosis* infection to SCC development in LV lesions remains limited, converging data from tuberculosis biology, chronic inflammation, and cancer immunology suggest several plausible, hypothesis-generating mechanistic pathways. Chronic LV lesions create a milieu of persistent inflammation, tissue damage, and scarring that can predispose to neoplastic transformation. Prolonged granulomatous inflammation generates reactive oxygen species and other free radicals that can induce DNA damage in adjacent epithelial cells. Over time, this mutagenic microenvironment may promote the development of dysplasia and ultimately SCC. Ultraviolet (UV) exposure is another contributing factor—many reported cases of SCC in LV occur on sun-exposed skin, implicating UV radiation in DNA damage (such as p53 tumor suppressor gene mutations) and local immunosuppression, thereby facilitating carcinogenesis. Chronic scarring and tissue remodeling in longstanding LV lesions are analogous to other settings of Marjolin’s ulcer, where scar tissue contributes to a tumorigenic milieu [36].

CTB, particularly its chronic forms such as LV, creates a proinflammatory microenvironment that fosters neoplastic transformation. Persistent colonization by *M. tuberculosis* leads to continuous engagement of pattern recognition receptors—most notably Toll-like receptors TLR2 and TLR4—on keratinocytes and macrophages [35,37]. This stimulation initiates downstream signaling cascades, including canonical activation of the IKK complex and nuclear translocation of NF-κB. Once activated, NF-κB promotes transcription of a broad repertoire of genes involved in inflammation (IL-6, TNF-α, IL-1β), resistance to apoptosis (Bcl-2), angiogenesis (VEGF), and cellular proliferation (COX-2), thus maintaining a self-sustaining inflammatory loop [38].

Simultaneously, IL-6 produced by macrophages and epithelial cells activates the JAK/STAT3 signaling pathway, culminating in persistent STAT3 phosphorylation and nuclear translocation. STAT3 enhances the expression of immunosuppressive (PD-L1, IL-10), anti-apoptotic (Bcl-xL, Bcl-2), and proliferative (Cyclin D1, c-Myc) mediators [39,40]. This immunoevasive and pro-proliferative milieu permits the survival of genetically unstable epithelial clones, further predisposing to carcinogenesis. Recent data also suggest that *M. tuberculosis* may modulate the Wnt/β-catenin signaling pathway, which could further influence epithelial cell fate, proliferation, and local immune responses in chronic cutaneous lesions [41].

Moreover, *M. tuberculosis*-induced stress signals, including ROS and inflammatory cytokines, activate MAPK signaling axes such as ERK, JNK, and p38 [42,43]. These pathways regulate transcription factors like AP-1 and ATF2, inducing genes that mediate keratinocyte proliferation (Cyclin D1, MMP-9), tissue remodeling, and inflammatory amplification (IL-6, TNF-α, COX-2) [44]. Prolonged activation of MAPKs thereby reinforces both epithelial hyperplasia and stromal reorganization [45].

Crucially, the chronic oxidative environment driven by macrophage-derived reactive oxygen and nitrogen species results in cumulative DNA damage within the surrounding epidermis. This activates DNA damage response kinases (ATM/ATR), leading to post-translational stabilization of the tumor suppressor p53 via phosphorylation and acetylation. Functional p53 coordinates transcriptional upregulation of cell cycle inhibitors (p21), DNA repair proteins (GADD45), and pro-apoptotic mediators (BAX, PUMA), thereby acting as a guardian of genomic integrity [46,47,48].

**Figure 3:**
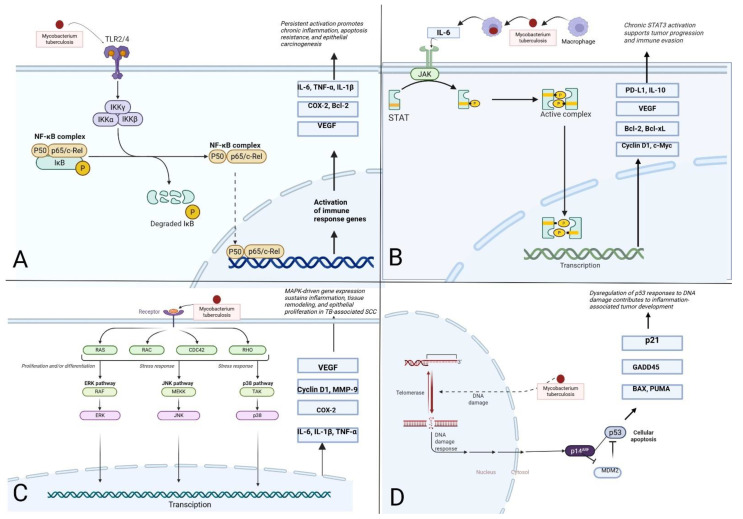
Schematic representation of signaling pathways potentially linking chronic CTB with squamous cell carcinoma (SCC) (Figure created with BioRender.com). (**A**) Activation of the NF-κB pathway via TLR2/4 engagement by *Mycobacterium tuberculosis* triggers IKK-mediated degradation of IκB and nuclear translocation of the NF-κB complex. This leads to transcription of pro-inflammatory, anti-apoptotic, and pro-angiogenic mediators such as IL-6, TNF-α, VEGF, and Bcl-2, contributing to sustained inflammation and epithelial transformation. (**B**) Chronic exposure to IL-6, secreted in response to *M. tuberculosis*, activates the JAK/STAT3 signaling cascade. Nuclear STAT3 drives expression of immunosuppressive and pro-proliferative genes (e.g., PD-L1, Bcl-xL, c-Myc), thereby promoting immune evasion and malignant progression. (**C**) Mycobacterial antigens and ROS initiate MAPK signaling (ERK, JNK, and p38), leading to activation of transcription factors such as AP-1. This enhances expression of genes involved in keratinocyte proliferation, matrix remodeling (MMP-9), and amplification of the inflammatory response. (**D**) Chronic oxidative stress induces DNA damage in keratinocytes, triggering activation of p53 via p14^ARF. Functional p53 promotes cell cycle arrest (p21), DNA repair (GADD45), and apoptosis (BAX, PUMA), but its dysregulation through mutations or MDM2 overexpression facilitates genomic instability and tumor development. (Figure created with BioRender.com).

However, under sustained inflammatory pressure, keratinocytes may acquire mutations in the TP53 gene, rendering p53 dysfunctional. Additionally, overexpression of endogenous inhibitors such as MDM2 may attenuate p53 activity [49]. The consequent failure to eliminate genetically damaged cells fosters clonal expansion and genomic instability—hallmarks of epithelial carcinogenesis. In this setting, loss of p53 checkpoint function synergizes with pro-oncogenic signals from NF-κB, STAT3, and MAPKs, potentially facilitating the evolution of SCC on a background of chronic tuberculosis [38,50]. A visual overview of the interconnected inflammatory and oncogenic pathways implicated in this process is provided in Figure 3.

As illustrated by this case and supported by literature, excision not only facilitates histopathologic assessment to rule out malignancy but also eliminates chronic inflammatory tissue that might otherwise harbor pre-neoplastic changes. In patients with recurrent or unresponsive lesions, surgical management can ensure disease resolution and prevent long-term complications. Such combined medical-surgical strategies exemplify the need for multidisciplinary care in CTB, integrating dermatology, infectious disease, pathology, and surgery to achieve optimal outcomes. Given the marked clinical polymorphism of lupus vulgaris and its frequent mimicry of other chronic inflammatory, infectious, and neoplastic dermatoses, a structured differential diagnostic approach remains essential in routine practice. A concise overview of key mimicking conditions and practical diagnostic considerations is summarized in Table 3.

In parallel, recent studies indicate that large language model-based and multimodal artificial intelligence systems may assist in dermatological image interpretation and clinical triage across a broad range of skin conditions. Such tools could, in the near future, support longitudinal assessment of chronic inflammatory skin diseases and help identify morphologic changes requiring histopathological verification, potentially shortening diagnostic delays in clinically polymorphic entities such as LV [59,60].

From a broader public health perspective, the relevance of this case extends beyond dermatology. According to the World Health Organization, TB once again became the world’s leading infectious killer in 2023, surpassing HIV/AIDS and COVID-19. This resurgence, driven by healthcare disruptions, drug resistance, and global migration, suggests that clinicians, even in low-incidence regions, will encounter extrapulmonary TB more often in coming years. Awareness of cutaneous manifestations is therefore essential to avoid diagnostic delay and prevent serious sequelae such as malignant transformation. In light of the discussed malignant potential of long-standing LV, a structured follow-up plan was established for the presented patient. Dermatologic surveillance is planned every 6 months, with focused examination of previously affected sites and post-surgical scars. Particular attention will be paid to new or evolving features, including induration, lesion enlargement, persistent ulceration, recurrent discharge, hyperkeratotic changes, or the development of pain. Any of these findings will prompt immediate histopathological evaluation.

While the case discussed in the present report provides insight into a complex and uncommon clinical scenario, several limitations should be acknowledged when interpreting the findings. First, it describes the clinical course and management of a single patient, which inherently limits the generalizability of the observations. Second, while anti-tubercular therapy for CTB is standardized, the role of surgical excision as an adjunctive measure is not explicitly addressed in existing treatment algorithms and remains based on individual clinical judgment. Nonetheless, it aims to contribute to clinical discussion and awareness of surgical intervention as a possible consideration in persistent or residual LV lesions.

In conclusion, SCC is a seldom but serious complication of LV. A heightened level of diagnostic vigilance is warranted in cases of chronic or treatment-refractory lesions. Surgical excision offers diagnostic clarity, definitive therapy, and may prevent malignant progression. In selected cases, this proactive approach should be considered part of optimal LV management. Timely recognition and appropriate management of LV are crucial to averting its uncommon yet serious complications.

## Figures and Tables

**Table 1:** Clinical forms of cutaneous tuberculosis and their key clinical and histopathological features.

Type of Cutaneous Tuberculosis	Route of Infection/ Immunological Context	Clinical Features	Histopathological Features	Typical Bacillary Load
Lupus vulgaris	Endogenous spread (hematogenous, lymphatic, contiguous) in patients with moderate to high immunity	Slowly enlarging reddish-brown plaques with central atrophy; “apple-jelly” color on diascopy; typically on head/neck	Well-formed epithelioid granulomas with or without caseation; epidermal atrophy or hyperkeratosis	Paucibacillary—AFB rarely seen
Scrofuloderma	Contiguous extension from underlying lymph node, bone, or joint TB; low to moderate immunity	Subcutaneous nodules that soften and ulcerate; draining sinus tracts with purulent discharge; common on neck	Tuberculoid granulomas with central caseation and abscess formation; frequent AFB positivity	Multibacillary
Tuberculosis verrucosa cutis	Exogenous inoculation in previously sensitized individuals with strong immunity	Verrucous, warty plaque with serpiginous borders, often on hands, knees, or feet	Pseudoepitheliomatous hyperplasia, hyperkeratosis, dermal granulomas with minimal necrosis	Paucibacillary
Tuberculous chancre	Exogenous inoculation in non-sensitized individuals; primary infection	Painless papule that ulcer with undermined edges; regional lymphadenitis common	Early neutrophilic infiltrate evolving to granulomas with caseation; abundant AFB in early stage	Multibacillary (early)
Tuberculosis orificialis	Autoinoculation from advanced pulmonary, GI, or GU TB in immunocompromised hosts	Painful ulcers at mucocutaneous junctions (oral cavity, anus, genitalia); shallow, undermined edges	Caseating granulomas rich in AFB	Multibacillary
Tuberculous gumma	Hematogenous spread during low immunity	Multiple cold abscesses that ulcerate and discharge caseous material	Diffuse necrosis with poorly formed granulomas; numerous bacilli	Multibacillary
Miliary cutaneous	Widespread hematogenous dissemination; severe immunosuppression	Multiple papules/pustules, some necrotic or umbilicated; systemic illness	Small necrotizing granulomas; abundant AFB	Highly multibacillary
Papulonecrotic tuberculid	Hypersensitivity reaction to *M. tuberculosis* antigens; strong immunity	Symmetrical necrotic papules on extensor surfaces; heal with varioliform scars	Leukocytoclastic vasculitis, fibrinoid necrosis, minimal granulomas; AFB absent	No viable bacilli
Lichen scrofulosorum	Hypersensitivity to distant focus of TB; mostly children and young adults	Tiny perifollicular papules in clusters on trunk or limbs	Perifollicular granulomas without caseation; AFB negative	No viable bacilli
Erythema induratum of Bazin	Hypersensitivity reaction, mainly in *women* with latent TB	Tender, recurrent nodules on posterior aspects of legs; may ulcerate	Lobular panniculitis with granulomatous vasculitis and fat necrosis	No viable bacilli

TB—tuberculosis; AFB—acid-fast bacilli; GI—gastrointestinal; GU—genitourinary.

**Table 2:** Reported cases of squamous cell carcinoma arising in chronic lupus vulgaris: clinical features, management, and outcomes.

Case Reference	Patient Demographics	LV Durtion (Years)	Site	Histology	Diagnosis	Treatment	Outcome
Yerushalmi et al. [27], 2002 (Israel)	47 M, Bedouin	40	Posterior neck	Mod-diff. SCC + granulomas	Biopsy; culture + for *M. tb*	Surgery, anti-TB, neck dissection, radiotherapy	Regional mets, treated, no recurrence
Gooptu et al. [28], 1998 (UK)	64 F, British	50	Face (cheek/jaw)	SCC in irradiated LV	Biopsy; Hx of Finsen lamp	Surgery	Local cure, no recurrence
Pătrașcu et al. [29], 2008 (Romania)	59 F, Romanian	57	Ear and cheek	Well-diff. SCC + nodal spread	Biopsy; lymph node histology	Excision, node dissection	Regional mets; under follow-up
Zawirska et al. [30], 2009 (Poland)	65 M, Polish	40	Face (cheeks)	SCC in exfoliative LV	Biopsy; granulomas + SCC	Surgery, anti-TB	Cured
Wulff-Woesten et al. [31], 2010 (Germany)	69 F, German	55	Thigh	SCC in LV with metastasis	Biopsy; nodal involvement	Surgery, chemo, anti-TB	Died of metastatic disease
Erdem et al. [32], 2011 (Turkey)	45 M, Turkish	20	Face/neck	Well-diff. SCC + granulomas	Biopsy	Surgery, anti-TB	Cured, no recurrence
Kumaran et al. [33], 2017 (India)	34 M, Indian	10	Beard area	Well-diff. SCC in MDR-TB LV	Biopsy; MDR culture	Surgery, 2nd-line TB drugs	Improved, no recurrence
Miyake et al. [26], 2017 (Japan)	71 M, Japanese	60	Face (nose/cheek)	SCC in chronic untreated LV	Biopsy	Surgical excision, anti-TB	Cured
Lin et al. [34], 2024 (China)	54 M, Chinese	20	Face (cheek)	Mod-diff. SCC + granulomas	Biopsy; T-SPOT TB+	Surgery, ALA-PDT, anti-TB	Complete remission
Chlebicka et al. [5], 2021 (Poland)	62 F, Polish	5	Hand (4th finger, interdigital)	SCC + granulomas; PCR+ for *M. tb*	Biopsy; PCR; imaging	Surgical excision with 0.5 cm margin, full-thickness graft	Cured, no metastasis, good functional result
Kanitakis et al. [35], 2006 (France)	70 F, French	45	Face	Well-diff. SCC in LV plaque	Histopathology	Surgery	Cured, no recurrence

LV—Lupus vulgaris; F—Female; M—Male; SCC—Squamous cell carcinoma; Mod-diff.—Moderately differentiated; Well-diff.—Well differentiated; PCR—Polymerase chain reaction; ALA-PDT—5-Aminolevulinic acid-based photodynamic therapy; *M. tb*—*Mycobacterium tuberculosis*; Hx—History; MDR-TB—Multidrug-resistant tuberculosis; TB—Tuberculosis.

**Table 3:** Selected differential diagnoses of LV—key clinical and diagnostic features.

Disease	Key Clinical, Histopathological, and Diagnostic Features (Including Biopsy Considerations) *	Source
LV (lupus vulgaris)	Chronic slowly progressive plaques or ulcers with scarring. Tuberculoid granulomas with epithelioid histiocytes and Langhans-type giant cells, usually without caseation; acid-fast bacilli typically absent. PCR may detect *M. tuberculosis* DNA. Biopsy from the active peripheral edge; consider split tissue for histopathology and mycobacterial PCR/culture. Initial evaluation includes chest imaging.	[51]
Sarcoidosis	Non-ulcerated plaques or nodules; non-caseating granulomas on histology; microbiologic studies negative. Biopsy from central lesional skin; chest imaging may reveal hilar lymphadenopathy; IGRA/TST often negative or indeterminate.	[52]
Cutaneous leishmaniasis	Chronic papules, nodules, or ulcers; travel or endemic exposure. Organisms detectable on smear, biopsy, or PCR. Biopsy typically obtained from the active lesion margin or ulcer base.	[53]
Deep mycoses (blastomycosis, sporotrichosis)	Verrucous or ulcerated lesions; suppurative or granulomatous inflammation. Fungal elements identifiable on PAS/GMS stains or culture; biopsy should include deep dermis.	[54]
Atypical (nontuberculous) mycobacterial infection	Chronic granulomatous lesions, often with exposure history. Histology overlaps with CTB. Biopsy from active edge recommended; unfixed tissue required for mycobacterial culture and PCR; IGRA typically negative.	[55]
SCC (squamous cell carcinoma)	Chronic indurated or ulcerated lesions with progressive growth and hyperkeratosis. Histopathology reveals epithelial dysplasia or invasive carcinoma. In long-standing inflammatory or scarred skin, SCC may arise as a Marjolin-type ulcer. Biopsy should target the most indurated or hyperkeratotic area to assess epithelial atypia and invasion.	[56]
Granuloma annulare	Annular plaques without ulceration; palisading granulomas with necrobiotic collagen and mucin. Biopsy from the active peripheral edge; microbiologic studies negative	[57]
Pyoderma gangrenosum	Rapidly progressive painful ulcers; pathergy phenomenon; neutrophilic dermatosis on histology without granulomas. Biopsy from lesion edge performed mainly to exclude infection or malignancy; cultures typically negative.	[58]

* In suspected infectious etiologies, tissue handling should allow for routine histopathology and, when feasible, unfixed material for microbiologic culture or molecular testing; formalin should be avoided for specimens intended for culture.

## Data Availability

The data underlying this article will be shared on reasonable request to the corresponding author.

## References

[1] WHO (2024). Tuberculosis Resurges as Top Infectious Disease Killer.

[2] WHO (2024). 2024 Global Tuberculosis Report.

[3] Sharma S.K., Mohan A. (2004). Extrapulmonary tuberculosis. Indian J. Med. Res..

[4] de Brito A.C., de Oliveira C.M.M., Unger D.A.A., de Bittencourt M.J.S. (2022). Cutaneous tuberculosis: Epidemiological, clinical, diagnostic and therapeutic update. An. Bras. Dermatol..

[5] Chlebicka I., Stefaniak A., Woźniak Z., Szepietowski J.C. (2019). Non-healing erythematous, ulcerated lesion on the hand: A quiz. Acta Derm.-Venereol..

[6] Bravo F.G., Gotuzzo E. (2007). Cutaneous tuberculosis. Clin. Dermatol..

[7] Zhang J., Fan Y.K., Wang P., Chen Q.Q., Wang G., Xu A.E., Hao F. (2018). Cutaneous tuberculosis in China—A multicentre retrospective study of cases diagnosed between 1957 and 2013. J. Eur. Acad. Dermatol. Venereol..

[8] Supekar B.B., Wankhade V.H., Singh R.P., Ghanate T.D., Bhat D. (2021). Clinical Spectrum of Cutaneous Tuberculosis in Central India: A Retrospective Study. Indian Dermatol. Online J..

[9] Couppoussamy K.I., Shanmugam S., Devanda R., Murugan R. (2024). Lupus vulgaris: A narrative review. Int. J. Dermatol..

[10] Nguyen K.H., Alcantara C.A., Glassman I., May N., Mundra A., Mukundan A., Urness B., Yoon S., Sakaki R., Dayal S. (2023). Cutaneous Manifestations of Mycobacterium tuberculosis: A Literature Review. Pathogens.

[11] Bayramgürler D., Sayan M., Aktürk A.Ş., Bilen N., Aslan N., Turan D. (2007). Disseminated lupus vulgaris presenting with different atypical lesions. J. Dermatol..

[12] Umapathy K.C., Begum R., Ravichandran G., Rahman F., Paramasivan C.N., Ramanathan V.D. (2006). Comprehensive findings on clinical, bacteriological, histopathological and therapeutic aspects of cutaneous tuberculosis. Trop. Med. Int. Health.

[13] Marcoval J., Alcaide F. (2013). Evolution of cutaneous tuberculosis over the past 30 years in a tertiary hospital on the European Mediterranean coast. Clin. Exp. Dermatol..

[14] Kaul S., Kaur I., Mehta S., Singal A. (2023). Cutaneous tuberculosis. Part I: Pathogenesis, classification, and clinical features. J. Am. Acad. Dermatol..

[15] Sethi A., Ataglance A. (2019). Chapter 157: Tuberculosis and Infections with Atypical Mycobacteria. Fitzpatrick’s Dermatology.

[16] Wang J., Liu J. (2024). Tuberculosis Verrucosa Cutis. N. Engl. J. Med..

[17] Padmavathy L., Lakshmana Rao L., Pari T., Ethirajan N., Krishnaswamy B. (2008). Lupus vulgaris and tuberculosis verrucosa cutis (TBVC)—A clinical, pathological and epidemiological study of 71 cases. Indian J. Tuberc..

[18] dos Santos J.B., Figueiredo A.R., Ferraz C.E., de Oliveira M.H., da Silva P.G., de Medeiros V.L.S. (2014). Cutaneous tuberculosis: Epidemiologic, etiopathogenic and clinical aspects—Part I. An. Bras. Dermatol..

[19] Fantoni O.J.J., Aryani I.A., Kurniawati Y., Pamudji R. (2021). Histopathological Features of Cutaneous Tuberculoid Granuloma Disorders. Biosci. Med. J. Biomed. Transl. Res..

[20] Chauhan V., Mahesh D.M., Panda P., Mahajan S., Thakur S. (2012). Tuberculosis cutis orificialis (TBCO): A rare manifestation of tuberculosis. J. Assoc. Physicians India.

[21] dos Santos J.B., Ferraz C.E., da Silva P.G., Figueiredo A.R., de Oliveira M.H., de Medeiros V.L.S. (2014). Cutaneous tuberculosis: Diagnosis, histopathology and treatment—Part II. An. Bras. Dermatol..

[22] Tirumalae R., Yeliur I.K., Antony M., George G., Kenneth J. (2014). Papulonecrotic tuberculid-clinicopathologic and molecular features of 12 Indian patients. Dermatol. Pract. Concept..

[23] Singal A., Kaur I., Pandhi D., Gandhi V., Jakhar D., Grover C. (2021). Clinico-epidemiological profile of lichen scrofulosorum: A 22-year, single-center, retrospective study. Int. J. Dermatol..

[24] Mascaró J.M., Baselga E. (2008). Erythema Induratum of Bazin. Dermatol. Clin..

[25] Kensler T.W., Egner P.A., Taffe B.G., Trush M.A. (1989). Role of free radicals in tumor promotion and progression. Prog. Clin. Biol. Res..

[26] Miyake T., Uhara H., Ishii N., Okuyama R. (2017). Squamous cell carcinoma arising from lupus vulgaris with a >60-year history. Int. Cancer Conf. J..

[27] Yerushalmi J., Grunwald M.H., Halevy D.H., Avinoach I., Halevy S. (1998). Lupus vulgaris complicated by metastatic squamous cell carcinoma. Int. J. Dermatol..

[28] Gooptu C., Marks N., Thomas J., James M.P. (1998). Squamous cell carcinoma associated with lupus vulgaris. Clin. Exp. Dermatol..

[29] Pǎtraşcu V., Georgescu C.V., Tǎnase L.E., Mogoantǎ S.S. (2008). Metastasized squamous cell carcinoma developed on lupus vulgaris. Rom. J. Morphol. Embryol..

[30] Zawirska A., Adamski Z., Stawicka E., Schwartz R.A. (2009). Cutaneous squamous cell carcinoma developing in lupus vulgaris exfoliativus persistent for 40 years. Int. J. Dermatol..

[31] Wulff-Woesten A., Hildebrandt U., Leverkus M., Ulrich J. (2010). Rapidly metastatic carcinoma in lupo in a patient with lupus vulgaris for more than 50 years. JDDG J. Ger. Soc. Dermatol..

[32] Erdem T., Aliaǧaoǧlu C., Yildirim M. (2011). Boyunda lupus vulgaristen gelişen skuamoz hücreli karsinom. Duzce Med. J..

[33] Kumaran M., Narang T., Jitendriya M., Tirumale R., Manjunath S., Savio J. (2017). Cutaneous squamous cell carcinoma in lupus vulgaris caused by drug resistant Mycobacterium tuberculosis. Indian Dermatol. Online J..

[34] Lin L., Huang Z., Xi B., Qin X., Yang K., Zhang R. (2024). Surgical Excision Combined with Photodynamic Therapy for Squamous Cell Carcinoma Arising in Lupus Vulgaris. Clin. Cosmet. Investig. Dermatol..

[35] Kanitakis J., Audeffray D., Claudy A. (2006). Squamous cell carcinoma of the skin complicating lupus vulgaris. J. Eur. Acad. Dermatol. Venereol..

[36] Ziegler A., Jonason A.S., Leffellt D.J., Simon J.A., Sharma H.W., Kimmelman J., Remington L., Jacks T., Brash D.E. (1994). Sunburn and p53 in the onset of skin cancer. Nature.

[37] Liu C.H., Liu H., Ge B. (2017). Innate immunity in tuberculosis: Host defense vs pathogen evasion. Cell. Mol. Immunol..

[38] Sharma N., Shariq M., Quadir N., Singh J., Sheikh J.A., Hasnain S.E., Ehtesham N.Z. (2021). Mycobacterium tuberculosis Protein PE6 (Rv0335c), a Novel TLR4 Agonist, Evokes an Inflammatory Response and Modulates the Cell Death Pathways in Macrophages to Enhance Intracellular Survival. Front. Immunol..

[39] Wang K., Karin M. (2015). Tumor-Elicited Inflammation and Colorectal Cancer. Advances in Cancer Research.

[40] Neumann J., Schaale K., Farhat K., Endermann T., Ulmer A.J., Ehlers S., Reiling N. (2010). Frizzled1 is a marker of inflammatory macrophages, and its ligand Wnt3a is involved in reprogramming Mycobacterium tuberculosis -infected macrophages. FASEB J..

[41] Villaseñor T., Madrid-Paulino E., Maldonado-Bravo R., Urbán-Aragón A., Pérez-Martínez L., Pedraza-Alva G. (2017). Activation of the Wnt pathway by Mycobacterium tuberculosis: A Wnt-Wnt Situation. Front. Immunol..

[42] Lim Y.J., Choi J.A., Lee J.H., Choi C.H., Kim H.J., Song C.H. (2015). Mycobacterium tuberculosis 38-kDa antigen induces endoplasmic reticulum stress-mediated apoptosis via toll-like receptor 2/4. Apoptosis.

[43] Hölscher C., Gräb J., Hölscher A., Müller A.L., Schäfer S.C., Rybniker J. (2020). Chemical p38 MAP kinase inhibition constrains tissue inflammation and improves antibiotic activity in Mycobacterium tuberculosis-infected mice. Sci. Rep..

[44] Wang W., Ning Y., Wang Y., Deng G., Pace S., Barth S.A., Menge C., Zhang K., Dai Y., Cai Y. (2022). Mycobacterium tuberculosis-Induced Upregulation of the COX-2/mPGES-1 Pathway in Human Macrophages Is Abrogated by Sulfasalazine. Front. Immunol..

[45] Bedoui S., Herold M.J., Strasser A. (2020). Emerging connectivity of programmed cell death pathways and its physiological implications. Nat. Rev. Mol. Cell Biol..

[46] Malik A.A., Sheikh J.A., Ehtesham N.Z., Hira S., Hasnain S.E. (2022). Can Mycobacterium tuberculosis infection lead to cancer? Call for a paradigm shift in understanding TB and cancer. Int. J. Med. Microbiol..

[47] Wang H., Zhang M., Xu X., Hou S., Liu Z., Chen X., Zhang C., Xu H., Wu L., Liu K. (2021). IKKα mediates UVB-induced cell apoptosis by regulating p53 pathway activation. Ecotoxicol. Environ. Saf..

[48] Liebermann D.A., Hoffman B. (2008). Gadd45 in stress signaling. J. Mol. Signal..

[49] Gdowicz-Kłosok A., Krześniak M., Łasut-Szyszka B., Butkiewicz D., Rusin M. (2025). Antibacterial Activity of the p53 Tumor Suppressor Protein-How Strong Is the Evidence?. Int. J. Mol. Sci..

[50] Chaussabel D., Semnani R.T., McDowell M.A., Sacks D., Sher A., Nutman T.B. (2003). Unique gene expression profiles of human macrophages and dendritic cells to phylogenetically distinct parasites. Blood.

[51] Kaul S., Jakhar D., Mehta S., Singal A. (2023). Cutaneous tuberculosis. Part II: Complications, diagnostic workup, histopathologic features, and treatment. J. Am. Acad. Dermatol..

[52] Koneti J., Cherukuri S.P., Gadde S., Kalluru R., Chikatimalla R., Dasaradhan T. (2022). Sarcoidosis and Its Dermatological Manifestations: A Narrative Review. Cureus.

[53] Reithinger R., Dujardin J.C., Louzir H., Pirmez C., Alexander B., Brooker S. (2007). Cutaneous leishmaniasis. Lancet Infect Dis..

[54] Carrasco-Zuber J.E., Navarrete-Dechent C., Bonifaz A., Fich F., Vial-Letelier V., Berroeta-Mauriziano D. (2016). Cutaneous Involvement in the Deep Mycoses: A Literature Review. Part I-Subcutaneous Mycoses. Actas Dermo-Sifiliogr..

[55] Winburn B., Sharman T. (2025). Atypical Mycobacterial Disease. StatPearls [Internet].

[56] Combalia A., Carrera C. (2020). Squamous Cell Carcinoma: An Update on Diagnosis and Treatment. Dermatol. Pract. Concept..

[57] Joshi T.P., Duvic M. (2022). Granuloma Annulare: An Updated Review of Epidemiology, Pathogenesis, and Treatment Options. Am. J. Clin. Dermatol..

[58] Maverakis E., Marzano A.V., Le S.T., Callen J.P., Brüggen M.C., Guenova E., Dissemond J., Shinkai K., Langan S.M. (2020). Pyoderma gangrenosum. Nat. Rev. Dis. Primers.

[59] Boostani M., Bánvölgyi A., Zouboulis C.C., Goldfarb N., Suppa M., Goldust M., Lőrincz K., Kiss T., Nádudvari N., Holló P. (2025). Large language models in evaluating hidradenitis suppurativa from clinical images. J. Eur. Acad. Dermatol. Venereol..

[60] Boostani M., Bánvölgyi A., Goldust M., Cantisani C., Pietkiewicz P., Lőrincz K., Holló P., Wikonkál N.M., Paragh G., Kiss N. (2025). Diagnostic Performance of GPT-4o and Gemini Flash 2.0 in Acne and Rosacea. Int. J. Dermatol..

